# Estimating and mitigating the effects of systemic low frequency oscillations (sLFO) on resting state networks in awake non-human primates using time lag dependent methodology

**DOI:** 10.3389/fnimg.2022.1031991

**Published:** 2023-01-19

**Authors:** Lei Cao, Stephen J. Kohut, Blaise deB. Frederick

**Affiliations:** ^1^Behavioral Neuroimaging Laboratory, McLean Hospital, Belmont, MA, United States; ^2^McLean Imaging Center, McLean Hospital, Belmont, MA, United States; ^3^Department of Psychiatry, Harvard Medical School, Boston, MA, United States; ^4^Opto-Magnetic Group, McLean Hospital, Belmont, MA, United States

**Keywords:** resting state networks, BOLD fMRI, functional connectivity analysis, systemic low frequency oscillations, physiological noise removal

## Abstract

**Aim:**

Resting-state fMRI (rs-fMRI) is often used to infer regional brain interactions from the degree of temporal correlation between spontaneous low-frequency fluctuations, thought to reflect local changes in the BOLD signal due to neuronal activity. One complication in the analysis and interpretation of rs-fMRI data is the existence of non-neuronal low frequency physiological noise (systemic low frequency oscillations; sLFOs) which occurs within the same low frequency band as the signal used to compute functional connectivity. Here, we demonstrate the use of a time lag mapping technique to estimate and mitigate the effects of the sLFO signal on resting state functional connectivity of awake squirrel monkeys.

**Methods:**

Twelve squirrel monkeys (6 male/6 female) were acclimated to awake scanning procedures; whole-brain fMRI images were acquired with a 9.4 Tesla scanner. Rs-fMRI data was preprocessed using an in-house pipeline and sLFOs were detected using a seed regressor generated by averaging BOLD signal across all voxels in the brain, which was then refined recursively within a time window of −16–12 s. The refined regressor was then used to estimate the voxel-wise sLFOs; these regressors were subsequently included in the general linear model to remove these moving hemodynamic components from the rs-fMRI data using general linear model filtering. Group level independent component analysis (ICA) with dual regression was used to detect resting-state networks and compare networks before and after sLFO denoising.

**Results:**

Results show sLFOs constitute ~64% of the low frequency fMRI signal in squirrel monkey gray matter; they arrive earlier in regions in proximity to the middle cerebral arteries (e.g., somatosensory cortex) and later in regions close to draining vessels (e.g., cerebellum). Dual regression results showed that the physiological noise was significantly reduced after removing sLFOs and the extent of reduction was determined by the brain region contained in the resting-state network.

**Conclusion:**

These results highlight the need to estimate and remove sLFOs from fMRI data before further analysis.

## 1. Introduction

Blood Oxygen Level Dependent (BOLD) resting state functional magnetic resonance imaging (rs-fMRI), which measures spontaneous low-frequency hemodynamic fluctuations in the brain during rest, is widely used to investigate functional organization of the brain. Because neuronal activity causes local changes in BOLD fMRI signal intensity, the temporal correlation of rs-fMRI signal fluctuations between brain regions is used to infer functional connectivity, i.e., neuronal communication between different regions, which can provide insight into large-scale functional dynamics of the brain (Friston, 1994; Biswal et al., 1995; Biswal, 2012). Two common approaches to determine functional connectivity are seed-based correlation mapping and independent component analysis (ICA; see Cole et al., 2010; Smitha et al., 2017). Through these methods, several distinct patterns of hemodynamic temporal coherence within different regions across the brain have been identified as resting-state networks (RSNs) (Biswal et al., 1995; Beckmann et al., 2005) and are thought to indicate synchronized neuronal activities within specific networks. These patterns have been extensively examined to understand the functional connectivity of each network, its relation to various behavioral, sensory, and cognitive processes, and how it may be altered during the progression of various disease states or in response to various stimuli.

One complication in the analysis and interpretation of rs-fMRI data is the existence of non-neuronal low frequency physiological noise. Previous studies have provided compelling evidence that systemic low frequency oscillations (sLFOs) constitute a significant fraction of the rs-fMRI signal in humans (i.e, 30–50% of the low frequency variance in gray matter (Tong and Frederick, 2010; Tong et al., 2015; Erdogan et al., 2016). Many attempts have been made to detect and limit the impact of low frequency physiological components on rs-fMRI data. For example, Birn et al. modeled respiration-induced low frequency oscillations (LFOs) from respiratory belt recordings and demonstrated their contribution to the default mode network (Birn et al., 2006, 2008). More recently, Tong et al. measured sLFOs in the periphery using near infrared spectroscopy (NIRS) and showed high correlation between sLFOs and several RSNs (Tong et al., 2013). Moreover, these results demonstrated good correspondence between the sLFO signal and rs-fMRI data throughout the brain, with a pattern of time delays suggesting that the sLFO signal was related to vascular anatomy (Tong and Frederick, 2010; Tong et al., 2011, 2016; Erdogan et al., 2016). This idea is further strengthened by the observation that such sLFO signals propagate within both the cerebral vasculature and throughout the body and have spatiotemporal patterns that reflect dynamic blood flow in the brain (Tong and Frederick, 2010; Erdogan et al., 2016; Tong et al., 2016).

The sLFO signal poses a problem for the interpretation of rs-fMRI data, as it occurs within the same low frequency band as the signal used to compute functional connectivity (i.e., 0.01–0.15 Hz). To address this problem, we have employed a time lag mapping methodology (Erdogan et al., 2016) by which the sLFO component of the brain's BOLD signal can be tracked in the absence of systemic recordings. Such a procedure provides a means to estimate the sLFO signal from the BOLD data itself, determine the voxel-specific time delay of the sLFO signal, quantify its contribution to the rs-fMRI signal, and remove it, mitigating its effect on the evaluation of RSNs. Further, since sLFOs travel with blood, the time when sLFOs reach each voxel can be interpreted as relative blood arrival time, yielding significant insight into cerebral hemodynamics (Tong et al., 2017, 2019).

The present study sought to demonstrate the implementation of this technique to estimate and mitigate the effects of the sLFO signal on the resting state networks of awake squirrel monkeys at ultra-high field (9.4 Tesla). Recently, Aso et al. reported a pattern of sLFOs in rs-fMRI data from rhesus monkeys that was similar to that shown in humans (Aso et al., 2020) suggesting that such procedures may help to decrease contamination of RSNs by non-neuronal sources across various species. The present study extends these findings in two important ways. First, we demonstrate that the contamination of resting state neuronal activation data by moving sLFO signals is seen even in small primates (this is the first study of this type in squirrel monkeys). Second, and perhaps more importantly, we demonstrate that unlike in human data, the sLFO signal is by far the largest source of low frequency variance in the fMRI data (over 60% in much of the gray matter, as much as double the contribution from neuronal activation). Therefore, though performing low frequency signal regression (either global signal regression or our time delayed method) is still a matter of debate and considered “optional” in human data; here we establish that it is essential in squirrel monkeys and, possibly, other animal species. Our findings are of clear translational value as many of the core RSNs that have been identified in humans (Smith et al., 2009, 2013) also have been identified in laboratory animal species including non-human primates (NHPs; Hutchison et al., 2011; Belcher et al., 2013; Yacoub et al., 2020; Liu et al., 2021) and rodents (Jonckers et al., 2011; Lu et al., 2012).

## 2. Methods

### 2.1. Subjects

Twelve experimentally naïve adolescent (~2.5 years of age) male and female squirrel monkeys (6 male; 6 female) (*Saimiri sciureus*) served as subjects. Subjects were housed in a temperature- and humidity-controlled vivarium with a 12-h light/dark cycle (0700–1900). Monkeys had unlimited access to water in the home cage and were maintained at approximate free-feeding weights with a nutritionally balanced diet of high protein chow (Purina Monkey Chow, St. Louis, MO). Fresh fruit and vitamins were provided as part of our environmental enrichment plan. The experimental protocol was approved by the Institutional Animal Care and Use Committee at McLean Hospital in a facility licensed by the US Department of Agriculture in accordance with guidelines provided by the Committee on Care and Use of Laboratory Animals of the Institute of Laboratory Animals Resources, Commission on Life Sciences.

### 2.2. Awake MRI training and acclimation

Extensive behavioral training was used to acclimate subjects to the MR procedures. Acclimation sessions, in which operant shaping techniques involving milk reinforcement were used to train subjects to move from the home-cage into a custom-designed MR-compatible chair, were typically conducted 5 days per week. Initially, subjects were trained to sit on their haunches in the prone position within the chair enclosure for brief, 5–10 min, sessions. This duration was gradually increased over several sessions to 30 min, after which, a helmet was introduced. The helmet was designed based on squirrel monkey anatomic images collected on our 9.4 Tesla Agilent/Varian MR system, 3-D printed (Silva et al., 2011), and lined with padding to limit motion and increase comfort for the subjects. The helmet, which also included a platform to position a transmit/receive surface coil, was mounted to the chair body with plastic screws. Once subjects were acclimated to the helmet, the session duration was further increased to 60 min. The final phase of acclimation involved moving subjects into a mock MRI bore housed within the laboratory. During mock scan sessions, recorded sounds from the scanner were played at decibels similar to those within the actual scanner (~90–100 dB). Vital signs (e.g., heart rate and oxygen saturation (SPO2); Nonin Model 7500FO, Plymouth, MN) were tracked and recorded at 5-min intervals throughout both training and actual MR sessions and subjects were continuously monitored through live video-feeds by a trained research assistant (VID-CAM-MONO-1 with SOF-842; Med-Associates, St. Albans, VT) (12M camera; MRC systems GmbH, Heidelberg, Germany).

### 2.3. Magnetic resonance imaging (MRI)

MRI scans were acquired using a 9.4 Tesla horizontal bore magnet system (Varian Direct Drive, Varian Inc, Palo Alto, CA, USA) running VnmrJ software (version 3.2A). An 11.6 cm inner diameter gradient was used for the present studies with maximum gradient strength of 40 G/cm and a transmit/receive surface coil was used for data collection. Following image localization and manual shimming, whole-brain gradient-echo EPI data were acquired at an isotropic resolution of 1.0 mm with a repetition rate of 1.5 s and echo time of 15 ms over a 30 min session. The fMRI sequence was run for 5 min without data capture prior to the acquisition to allow the gradients to come to thermal equilibrium and minimize artifactual drift.

### 2.4. MRI data processing

fMRI preprocessing was conducted with the FSL software library (FMRIB, Oxford University, UK) (Smith et al., 2004; Woolrich et al., 2009; Jenkinson et al., 2012; Kohut et al., 2020). The first 100 volumes and last 200 volumes of the datasets were removed to minimize motion effects, leaving 900 volumes for analysis. Intensity spiking was detected and mitigated using an open-source program (https://github.com/bbfrederick/spikefix) with a percentage threshold of 2 and a voxel threshold of 129 (defined as 3 median average deviates greater than the median number of voxels exceeding the percentage threshold). The de-spiked images were then motion corrected and high pass filtered with a 100 s cutoff. For registration to standard space, functional volumes were aligned to the VALiDATe (Gao et al., 2014: Schilling et al., 2017) T2w template through a 12 DOF affine transformation followed by adjustment of nonlinear distortion fields using the jip analysis toolkit (www.nitrc.org/projects/jip). The VALiDATe brain mask was subsequently applied to perform final skull stripping. For quality assurance, scan metrics were assessed using MRIQC (Esteban et al., 2017), and group mean motion statistics were computed for the dataset.

### 2.5. sLFO characterization and removal

Systemic low frequency oscillation was characterized using rapidtide, an open-source package implementing the Regressor Interpolation at Progressive Time Delays (RIPTiDe) method (Frederick, 2016). Details of the RIPTiDe procedure have been described previously (Erdogan et al., 2016; Tong et al., 2019). Briefly, the default Rapidtide parameters were used with the exception of some modifications noted below. First, the resting state fMRI data was filtered to the low frequency band (0.01–0.15 Hz) and a Gaussian spatial filter with a 0.75 mm kernel width was applied to smooth the data, reflecting the smaller brain size of the squirrel monkey. A seed regressor was generated by averaging BOLD signal across all voxels in the brain, and recursively refined in three passes by calculating the relative arrival time of the regressor in each voxel by fitting the peak of the cross-correlation function within a time window of −16 to 12 s relative to the mode of the delay times (NB: the time window used is larger than the expected delay range to allow for better fitting of the relatively broad correlation peaks. The search range parameter is an operational tuning parameter of the Rapidtide program and does not determine the range of delays obtained. The search range used here was empirically determined from multiple runs), time-shifting each voxel's time course to align with the regressor, and then using principal component analysis to extract a signal representing 80% of the variance of all shifted BOLD voxels. The refined regressor was cross-correlated with the time course in each voxel, and the peak of the cross-correlation was fit with a Gaussian function to calculate the maximum correlation coefficient and the relative arrival time of sLFO in each voxel. If no peak could be uniquely determined, the delay and maximum correlation value were set to zero; however, the SNR was sufficiently high after spatial filtering that there were very few voxels without fittable peaks. In contrast to humans, the blood arrival time distribution was found to be bimodal. Therefore, the initial moving regressor was calculated and refined using only gray matter voxels, as the blood signal is stronger and has more uniform delays in gray matter in squirrel monkeys, and the “pickleft” flag was used to restrict refinement to voxels in the earlier delay time peak. The relative arrival time was defined as the time when maximum cross-correlation was reached. It is important to note that since we do not have an absolute reference for zero arrival time, zero is set arbitrarily to the peak of the arrival time distribution histogram, i.e., the mode. The refined regressor was then time-shifted by this relative arrival time to generate voxel-wise regressors to remove the moving hemodynamic component from the functional data by means of general linear model. The VALiDATe gray matter mask was supplied to restrict the calculation to gray matter only.

### 2.6. Pseudoperiodicity

While using a frequency range up to 0.15 Hz (~6.67 s cycle time) does lead to the possibility that the moving regressor will be pseudoperiodic, this is unavoidable, as this is the range in which neuronally driven hemodynamic fluctuations are found. Pseudoperiodicity in the sLFO waveform is always a concern in performing rapidtide analysis when unambiguous delay estimation is the goal. Fortunately, this is not as large a problem as it may seem, particularly in this instance. First, because the sLFO signal is essentially random, usually with a fairly flat frequency spectrum, it is usually *not* pseudoperiodic, and as long as any “dominant” frequency is below ~0.06 Hz, correlation sidelobes will not occur within the peak search range. Second, rapidtide checks the regressor for pseudoperiodicity, and implements a number of mitigation strategies, including voxelwise peak shifting by the pseudoperiod to remove spatial discontinuities in the delay map, and notch filtering out the dominant periodic frequency during delay estimation. Finally, in the case of denoising, as we are doing here, if there are multiple indistinguishable peaks in the cross-correlation, then which one we use is not important, as we will remove essentially the same noise signal in each case (this is not the case when we are estimating delay for its own sake).

### 2.7. Independent component analysis and dual regression

Group level time concatenated independent component analysis (ICA) and dual regression were conducted within FSL to evaluate the performance of the noise reduction method. Initially, a series of group ICAs were computed with the non-denoised dataset, with varied dimensionality (20, 25, 30, 35, 40, 45, 50), to determine the optimal number of independent components (ICs). The optimal number of ICs was chosen based on sufficient separation from noise and reasonable merging of the same resting state networks and is consistent with previously published reports in animal subjects (e.g., Hutchison et al., 2011; Jonckers et al., 2011; Belcher et al., 2013). The group IC maps estimated from group ICA with the dimensionality of 25 were then used as template in the following dual regression. The 25 component ICA was repeated on the rapidtide-denoised dataset, and the components in the denoised ICA were matched to their non-denoised equivalents using PICAchooser (Frederick, 2020).

The dual regression was carried out in two steps. The group spatial IC template was first regressed into both the non-denoised dataset and the denoised dataset to compute subject-specific timecourses. Subject-specific spatial ICs were then identified by regressing subject-specific timecourses against each subject's own fMRI data. Next, permutation-based non-parametric testing was implemented to quantify the difference in ICs before and after noise reduction; 5,000 permutations were used in the analysis and the resulting statistical maps were thresholded at *p* ≤ 0.05 (threshold-free cluster enhancement (TFCE) corrected). To visualize the distribution of sLFO relative arrival time within each IC, a probability density map was plotted for each IC.

## 3. Results

### 3.1. sLFO signal estimation with RIPTiDe

**Figure 1:**
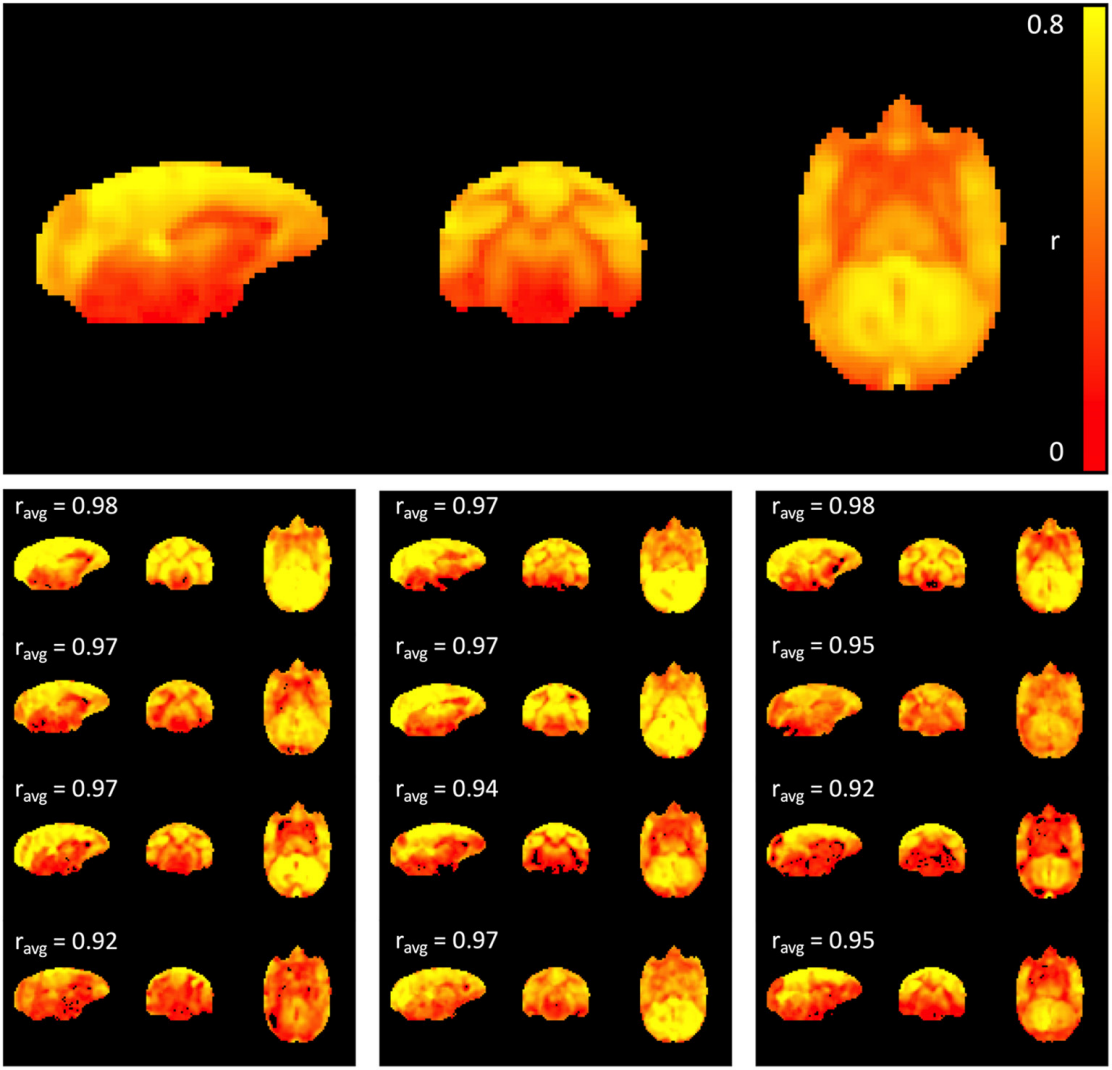
Maximum correlation coefficient map. The top panel shows the average map across all 12 subjects, while the maps at the bottom represent subject-specific maximum correlation coefficient maps. The percentage of variance explained by the time shifted LFO regressor in each voxel is 100 * (correlation coefficient)^2^. Cross-correlation between each individual map and the average map was performed, and the correlation coefficient is indicated above each individual map; note that correlation values are strong across all subjects (>0.9).

The top panel of Figure 1 shows the average maximum correlation coefficient map of all 12 subjects. High correlation values were observed, primarily within gray matter, suggesting that sLFO constitute a significant portion of the fMRI signal within the low frequency range. The individual maximum correlation coefficient maps for each subject in the lower panels show that the pattern is consistent across individual subjects.

**Figure 2:**
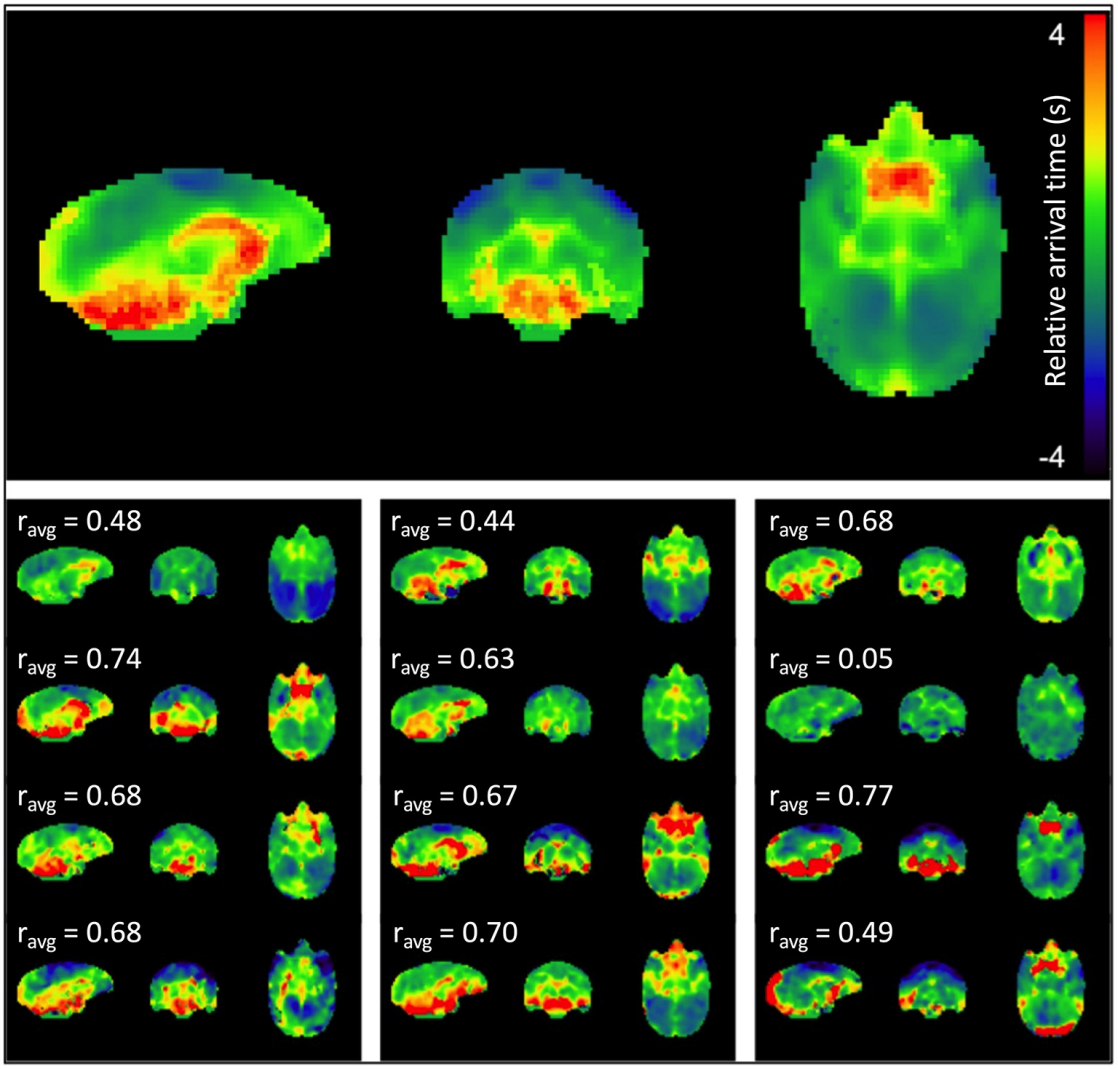
sLFO arrival time maps. The mean arrival time map for the group is shown at the top, followed by subject-specific individual arrival time maps. Delays are in seconds relative to the peak of the voxelwise delay histogram. Cross-correlation between each individual map and the average map was performed, and the correlation coefficient is indicated above each individual map. The majority of the correlations (8 of 12 subjects) were moderate (0.5–0.7) to strong (≥0.7).

Figure 2 displays the sLFO relative arrival time maps (group average in the top panel, individual maps below). The regions of early arrival are shown in cool colors (blue, light blue and green), while regions of late arrival are shown in warm colors (red and yellow). The map clearly depicts a consistent pattern of blood circulation in squirrel monkey brain; sLFOs arrive early in bilateral motor and somatosensory cortex where middle cerebral arteries reside, while those regions with later arrival of sLFO appeared mainly in regions close to the vascular drainage system, i.e., ventricles and cerebellum. White matter also has generally later arrival times than gray matter. The sLFO circulation pattern is consistent across subjects, with varied sLFO arrival time, with differences most likely due to individual variation in the vascular anatomy. Overall, these results support the contention that the sLFO signal is embedded in the underlying physiological circulation and is consistent with blood flow mapping in humans using sLFO delays (Erdogan et al., 2016), which have been cross validated with dynamic susceptibility mapping (Erdogan et al., 2016).

### 3.2. Group level independent component analysis and dual regression

**Figure 3:**
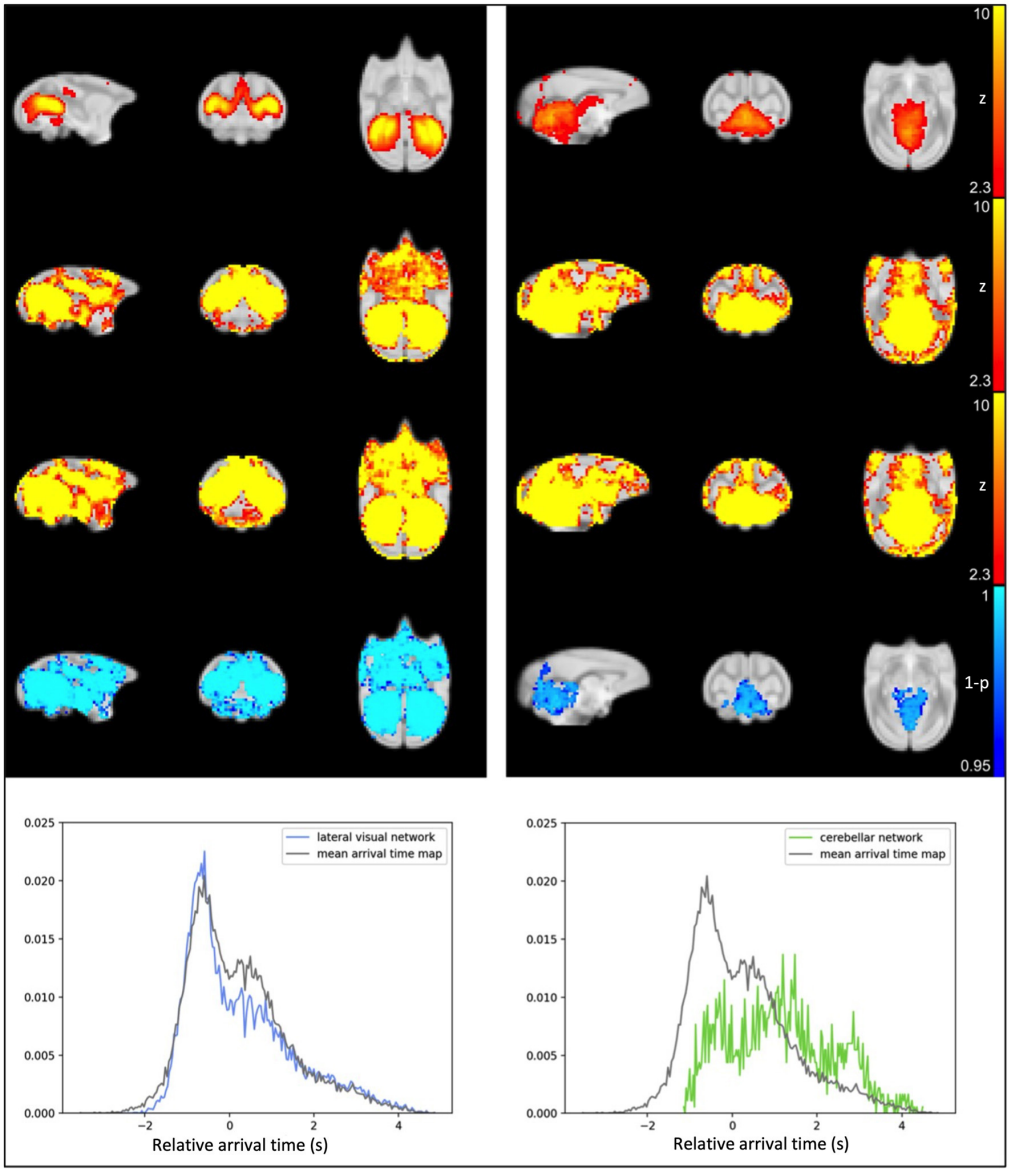
Results from IC1, the lateral visual **(left)** and IC14, the cerebellar network **(right)**. **Top row:** IC computed from original data; **second row:** averaged spatial ICs of non-denoised dataset; **third row:** averaged spatial ICs of denoised dataset; **fourth row:** statistical map representing significant reduction in IC after sLFO removal (TFCE-corrected), the number of significant voxels is also calculated and indicated above the statistical map; **fifth row:** probability density map showing number of voxels within IC at different sLFO arrival time.

After visual inspection of a series of group IC maps with different dimensionality, 25 spatial IC maps computed by group ICA of the original data were used as the template for dual regression and subsequent statistical analysis to compare the networks before and after the application of rapidtide denoising. Figure 3 shows the comparison results for two of the representative resting state networks; lateral visual network and cerebellar network. For referencing purposes, the IC templates (original resting state network) are shown in the first row of Figure 3. The second row represents group average of spatial ICs of non-denoised dataset, and the third row displays group mean spatial ICs for the denoised dataset. Group comparison results through permutation testing are presented in row 4. The lateral visual network exhibits a wide distribution of significant noise reduction; in contrast, the cerebellar network exhibits very localized noise reduction. Based on their sLFO arrival time distribution (row 5), the voxels within the lateral visual network have similar range of sLFO relative arrival times as the voxels within the whole brain (the arrival time distribution largely overlaps with that of the whole brain sLFO), hence rapidtide denoising affects multiple voxels across the whole brain. The voxels within the cerebellar network, however, have relatively later sLFO arrival time (the peak occurs several seconds after the peak of whole brain distribution); thus, noise reduction only exists in voxels with later sLFO arrival time. This relationship holds for most of the networks; the number of significantly changed voxels tends to increase as the center of mass (COM) of the delay distribution within the IC moves farther from the overall COM of delays throughout the brain. In fact, 16 of the 25 ICs showed >25% of voxels and 8 of 25 ICs showed >50% of voxels in the brain change significantly after denoising (see Supplementary Figures 1A–G, Supplementary Table 1). It's important to recognize that ICs are thresholded for display to show only voxels that meet a level of significance, but the IC contains continuous spatial patterns that extend throughout the brain and exist both within, and outside, of the thresholded z-maps. This is described in greater detail in the Supplementary material, along with depictions of all the significant networks before and after denoising.

## 4. Discussion

The present study demonstrates that time lag mapping can be successfully applied to non-human primate fMRI data for visualizing spatiotemporal patterns of sLFOs. This finding is consistent with previous reports in human subjects (Tong et al., 2017). Furthermore, the delay map clearly resembles the patterns of cerebral blood circulation, implying a hemodynamic, rather than neuronal, origin. The sLFO relative arrival time patterns, while generally similar, vary across individuals since they have different vascular anatomy. This yields new insight into the circulatory architecture of non-human primates, as well as information on individual differences which may be of use to assess neurovascular health.

Perhaps of more interest to those interested in neuronal connectivity, however, is the magnitude of this signal component. The correlation maps indicate that up to 64% of the voxel-wise BOLD signal variance (100 × the square of the correlation coefficient) was explained by sLFOs. In contrast, gray matter sLFO variance in humans is predominantly < 50% (Erdogan et al., 2016). Therefore, it is even more important to remove this heterogeneous signal of non-neuronal origin in squirrel monkeys. In an attempt to better illustrate the effect of sLFO contamination on resting state networks, we performed an ICA analysis to extract resting-state networks using the same rs-fMRI data with and without denoising. Although we tuned the number of components to maximize the separation of noise while still maintaining the networks of interest, each component still contains significant low frequency physiological noise. This will negatively affect subsequent statistical analysis and interpretation. Since up to ~2/3 of the gray matter signal variance in the low frequency band used to estimate neuronal connectivity carries no relevant information about neuronal activity, if these signals are not removed, they will bias connectivity measures toward hemodynamic factors (i.e., areas where blood coincidentally arrives at similar times will appear to be functionally connected). In pharmacological challenges, which can have profound effects on both neuronal and hemodynamic function, the inability to separate these effects will significantly limit the validity of connectivity changes as a tool for assessing neuronal effects. In the current study, the RSNs computed from denoised data and the dual regression results comparing RSNs before and after denoising showed that the physiological noise was significantly reduced after removing sLFOs, and the extent of reduction is related to the brain region contained in the IC. More specifically, if ICs contain brain regions that have a similar sLFO arrival time as the majority of brain regions, they will have broad reductions after denoising. Contrarily, if ICs contain voxels with sLFO relative arrival times that differ significantly with the majority of the voxels within the whole brain, more localized reductions could be observed. These results have further demonstrated the need to remove sLFOs before analyzing rs-fMRI data.

This analysis demonstrates the rapidtide package (Frederick, 2016), provides a simple set of open-source turnkey tools to perform this retrospective denoising in non-human primate data. It is easily applied retrospectively to existing datasets, as it can extract sLFO regressors directly from the fMRI data itself without the need of external recordings, and it removes significant hemodynamic noise contamination from resting state data without the pitfalls of global signal regression (Erdogan et al., 2016). Therefore, we suggest incorporating rapidtide denoising in non-human primate fMRI data as a standard step in the preprocessing pipeline.

## Data availability statement

The raw data supporting the conclusions of this article will be made available by the authors, without undue reservation.

## Ethics statement

The animal study was reviewed and approved by McLean Hospital Institutional Animal Care and Use Committee.

## Author contributions

Participated in research design and wrote or contributed to the writing of the manuscript: LC, SK, and BF. Conducted experiments: SK. Contributed new reagents or analytic tools: BF. Performed data analysis: LC and BF. All authors contributed to the article and approved the submitted version.
