# Supplementary material for: Estimating and mitigating the effects of systemic low frequency oscillations (sLFO) on resting state networks in awake non-human primates using time lag dependent methodology
Source: Front Neuroimaging. 2023 Jan 19;1:1031991. doi: 10.3389/fnimg.2022.1031991 (PMC10406257; doi:10.3389/fnimg.2022.1031991)
Supplement: Supplementary file 1 [file Presentation_1.PPTX]

## Slide 1
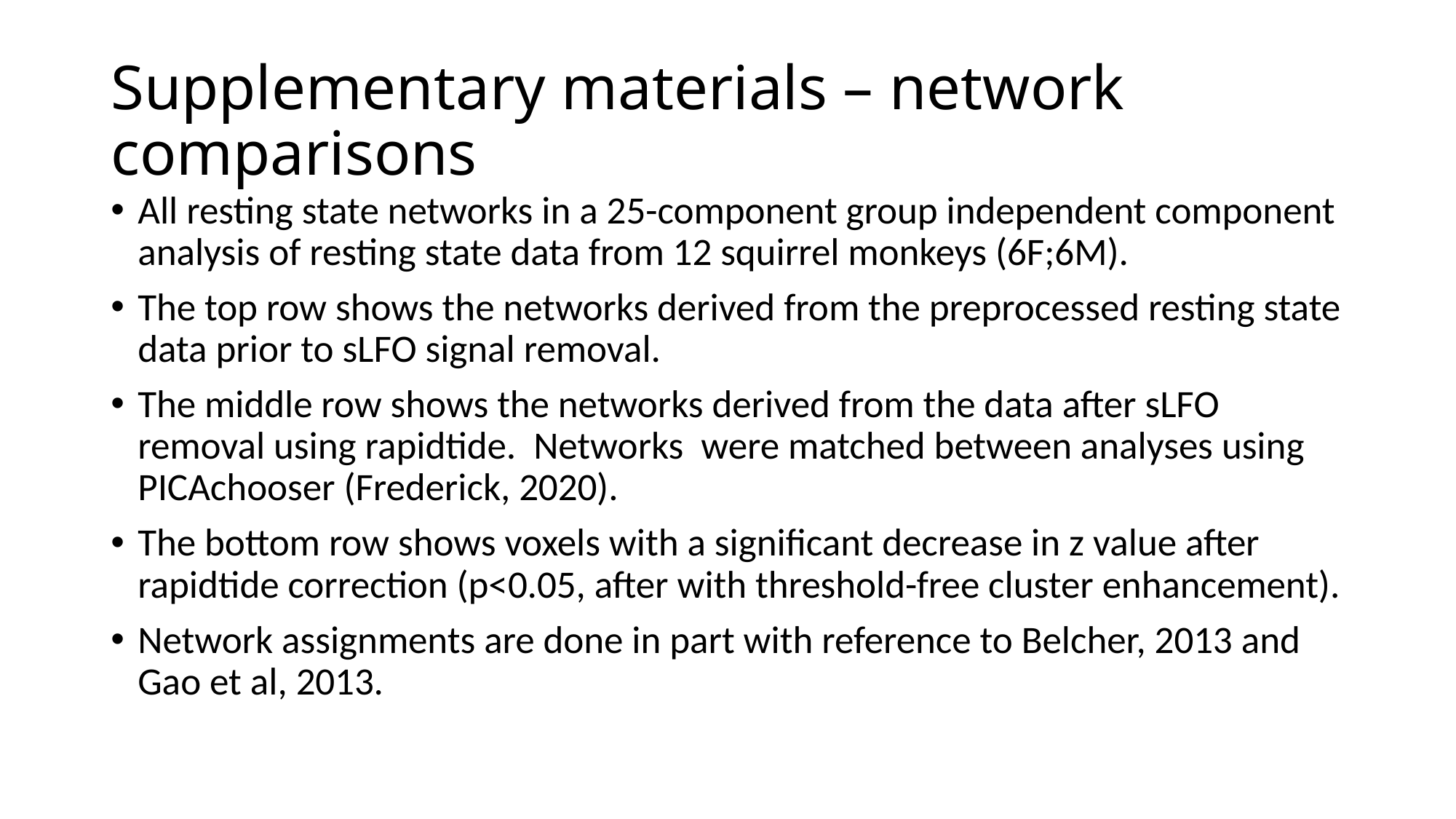

# Supplementary materials – network comparisons
All resting state networks in a 25-component group independent component analysis of resting state data from 12 squirrel monkeys (6F;6M).
The top row shows the networks derived from the preprocessed resting state data prior to sLFO signal removal.
The middle row shows the networks derived from the data after sLFO removal using rapidtide. Networks were matched between analyses using PICAchooser (Frederick, 2020).
The bottom row shows voxels with a significant decrease in z value after rapidtide correction (p<0.05, after with threshold-free cluster enhancement).
Network assignments are done in part with reference to Belcher, 2013 and Gao et al, 2013.

## Slide 2
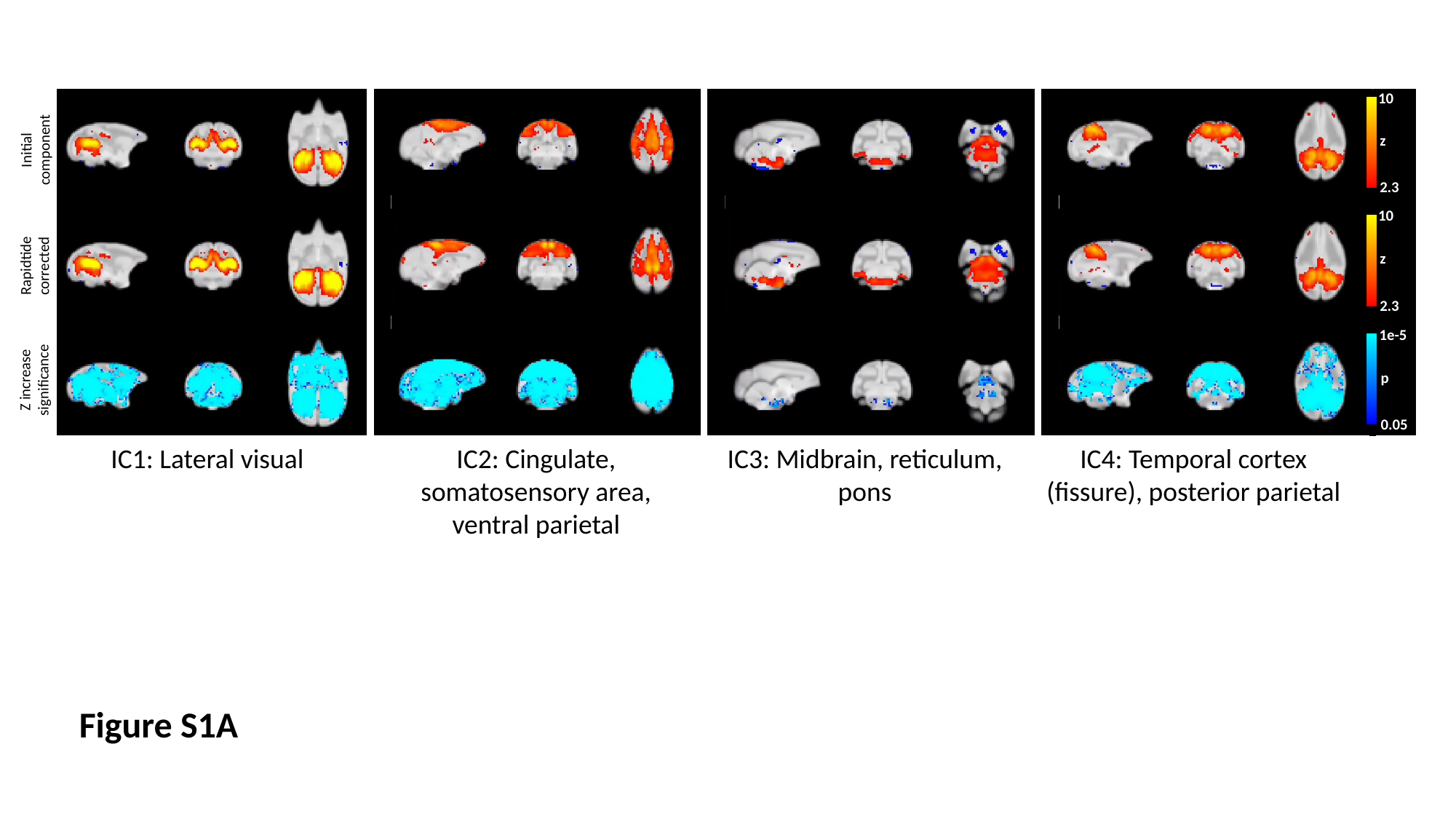

10
z
2.3
10
z
2.3
1e-5
p
0.05
Initial
component
Rapidtide
corrected
Z increase
significance
IC1: Lateral visual
IC2: Cingulate, somatosensory area, ventral parietal
IC3: Midbrain, reticulum, pons
IC4: Temporal cortex (fissure), posterior parietal
Figure S1A

## Slide 3
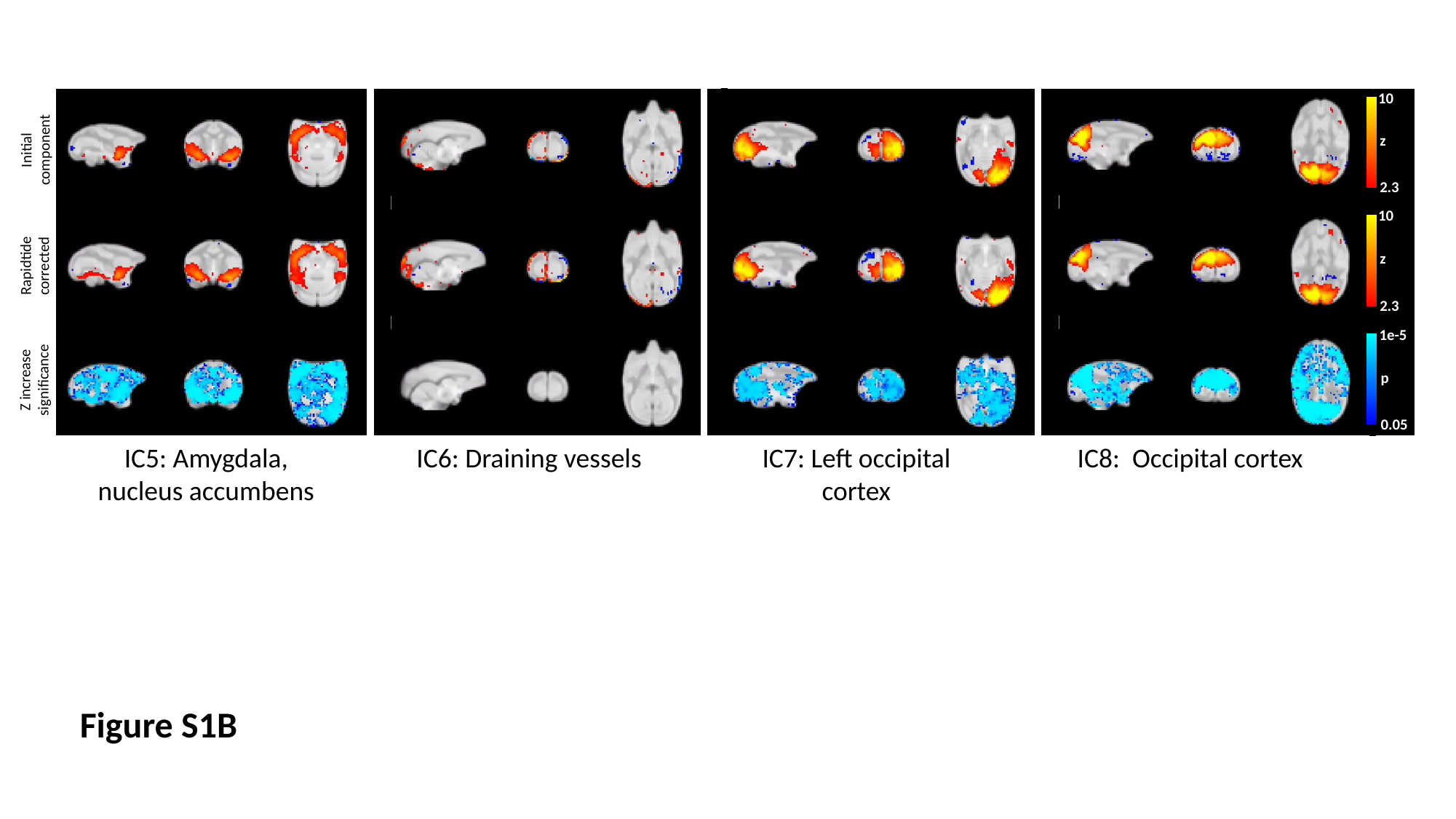

10
z
2.3
10
z
2.3
1e-5
p
0.05
Initial
component
Rapidtide
corrected
Z increase
significance
IC5: Amygdala, nucleus accumbens
IC6: Draining vessels
IC7: Left occipital cortex
IC8: Occipital cortex
Figure S1B

## Slide 4
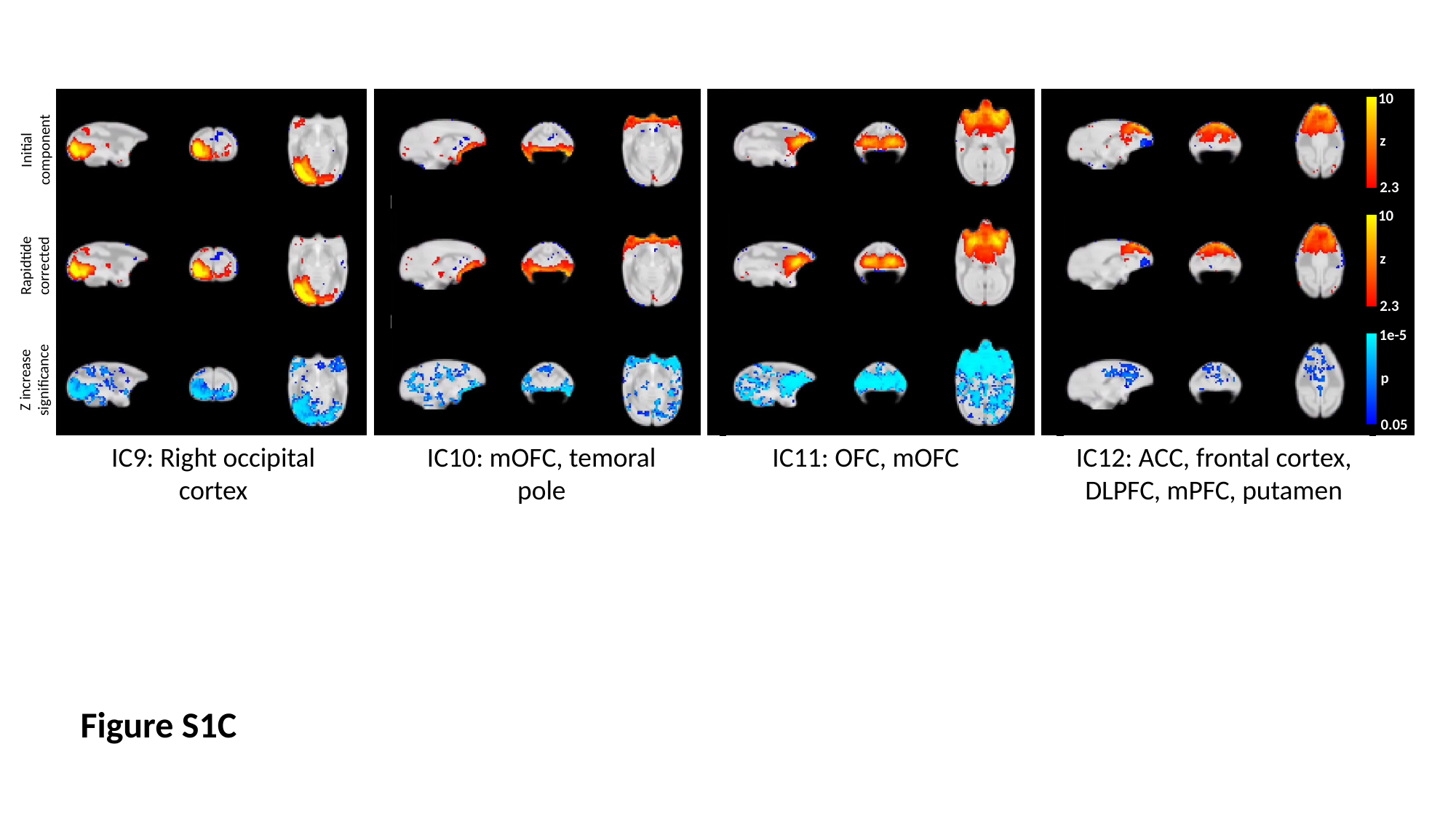

10
z
2.3
10
z
2.3
1e-5
p
0.05
Initial
component
Rapidtide
corrected
Z increase
significance
IC9: Right occipital cortex
IC10: mOFC, temoral pole
IC11: OFC, mOFC
IC12: ACC, frontal cortex, DLPFC, mPFC, putamen
Figure S1C

## Slide 5
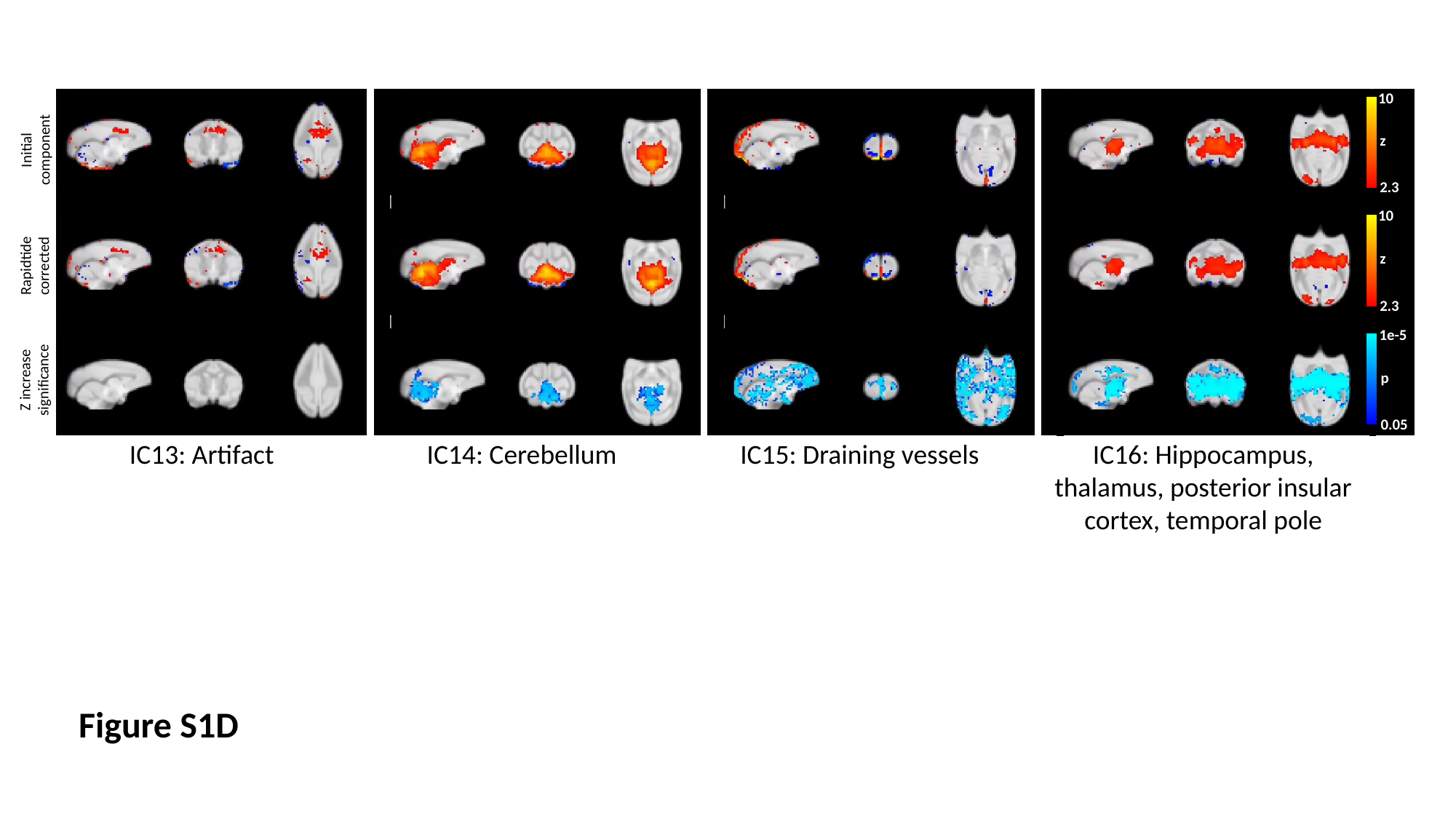

10
z
2.3
10
z
2.3
1e-5
p
0.05
Initial
component
Rapidtide
corrected
Z increase
significance
IC13: Artifact
IC14: Cerebellum
IC15: Draining vessels
IC16: Hippocampus, thalamus, posterior insular cortex, temporal pole
Figure S1D

## Slide 6
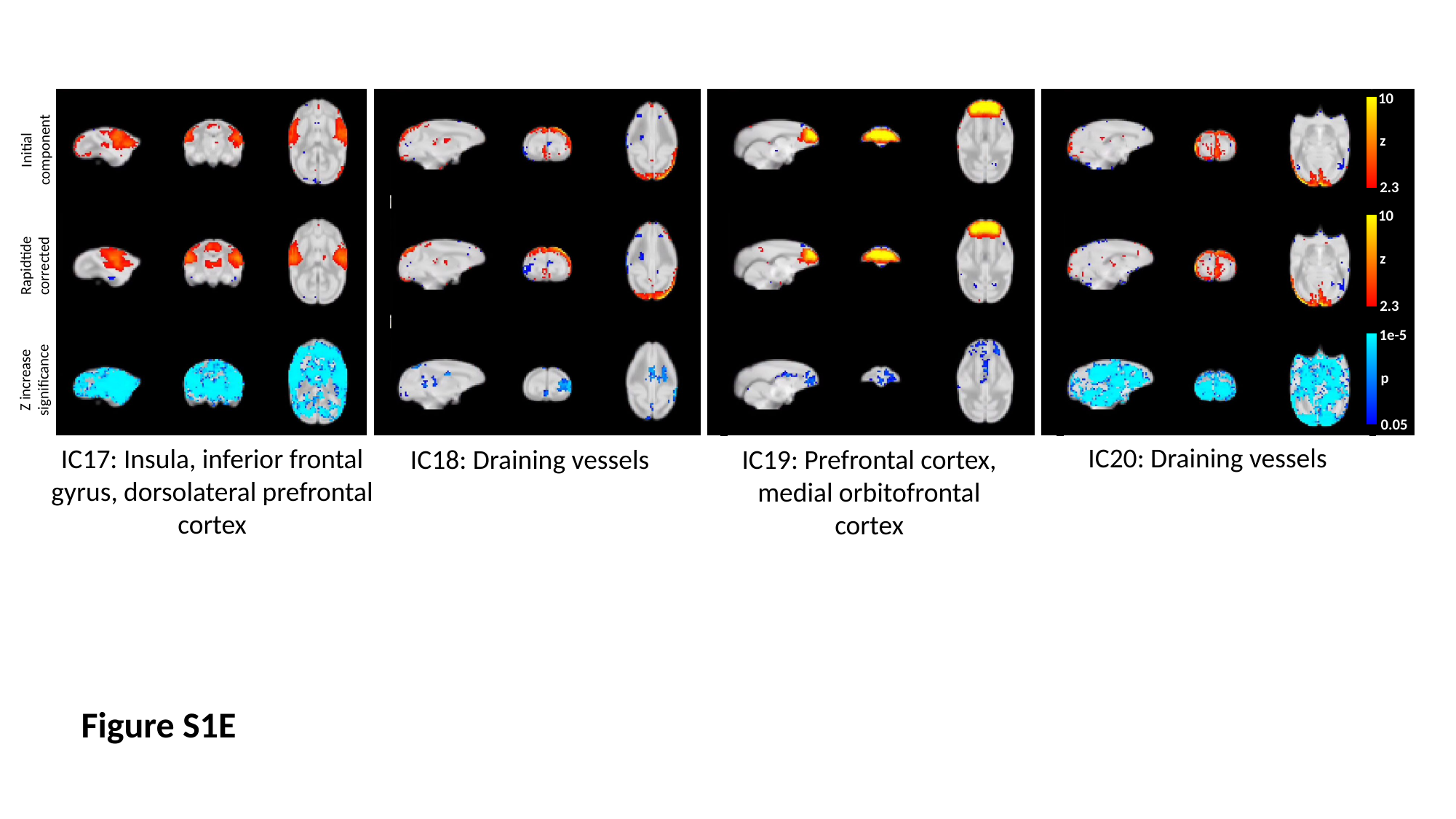

10
z
2.3
10
z
2.3
1e-5
p
0.05
Initial
component
Rapidtide
corrected
Z increase
significance
IC20: Draining vessels
IC17: Insula, inferior frontal gyrus, dorsolateral prefrontal cortex
IC18: Draining vessels
IC19: Prefrontal cortex, medial orbitofrontal cortex
Figure S1E

## Slide 7
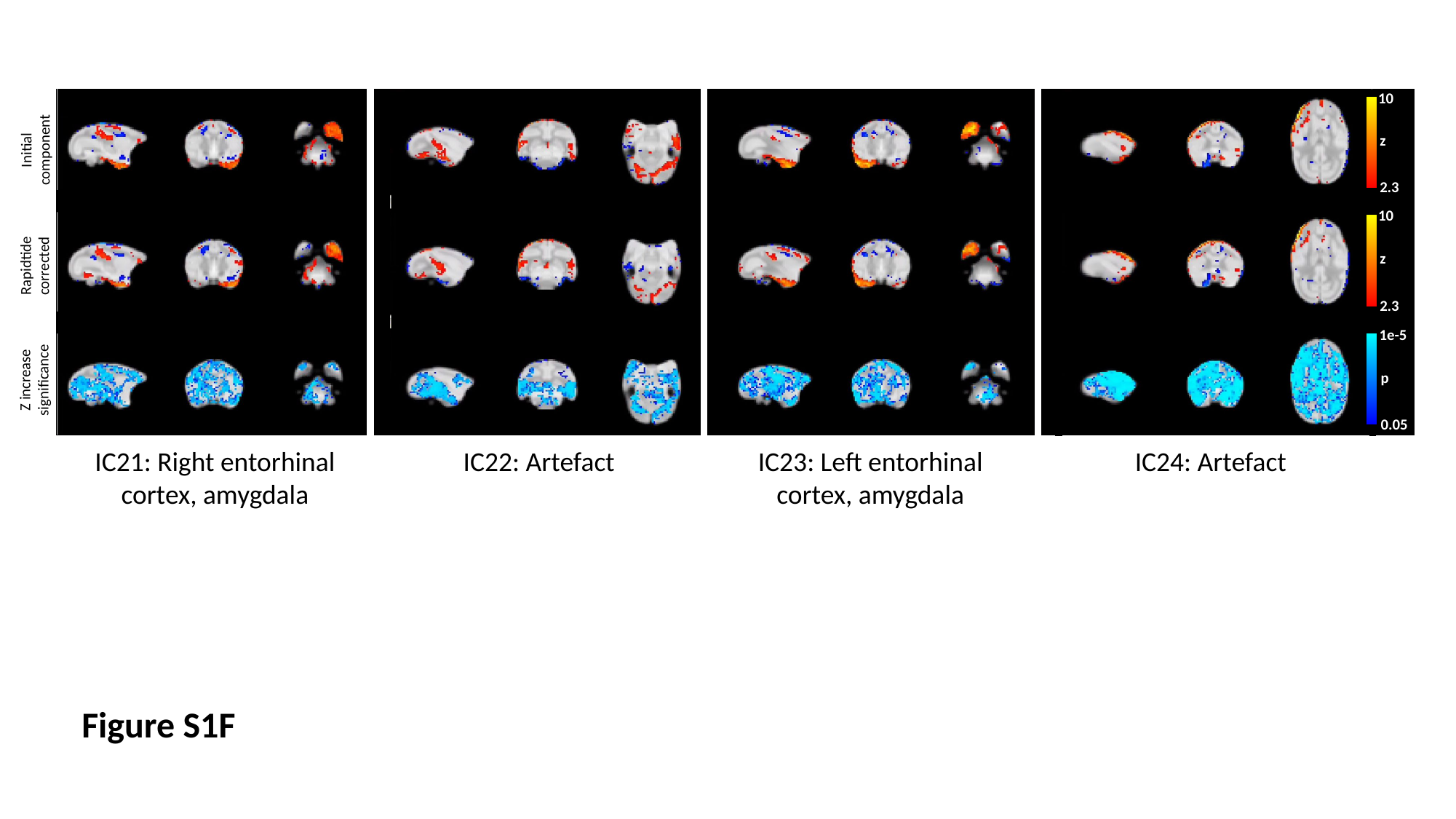

10
z
2.3
10
z
2.3
1e-5
p
0.05
Initial
component
Rapidtide
corrected
Z increase
significance
IC22: Artefact
IC23: Left entorhinal cortex, amygdala
IC24: Artefact
IC21: Right entorhinal cortex, amygdala
Figure S1F

## Slide 8
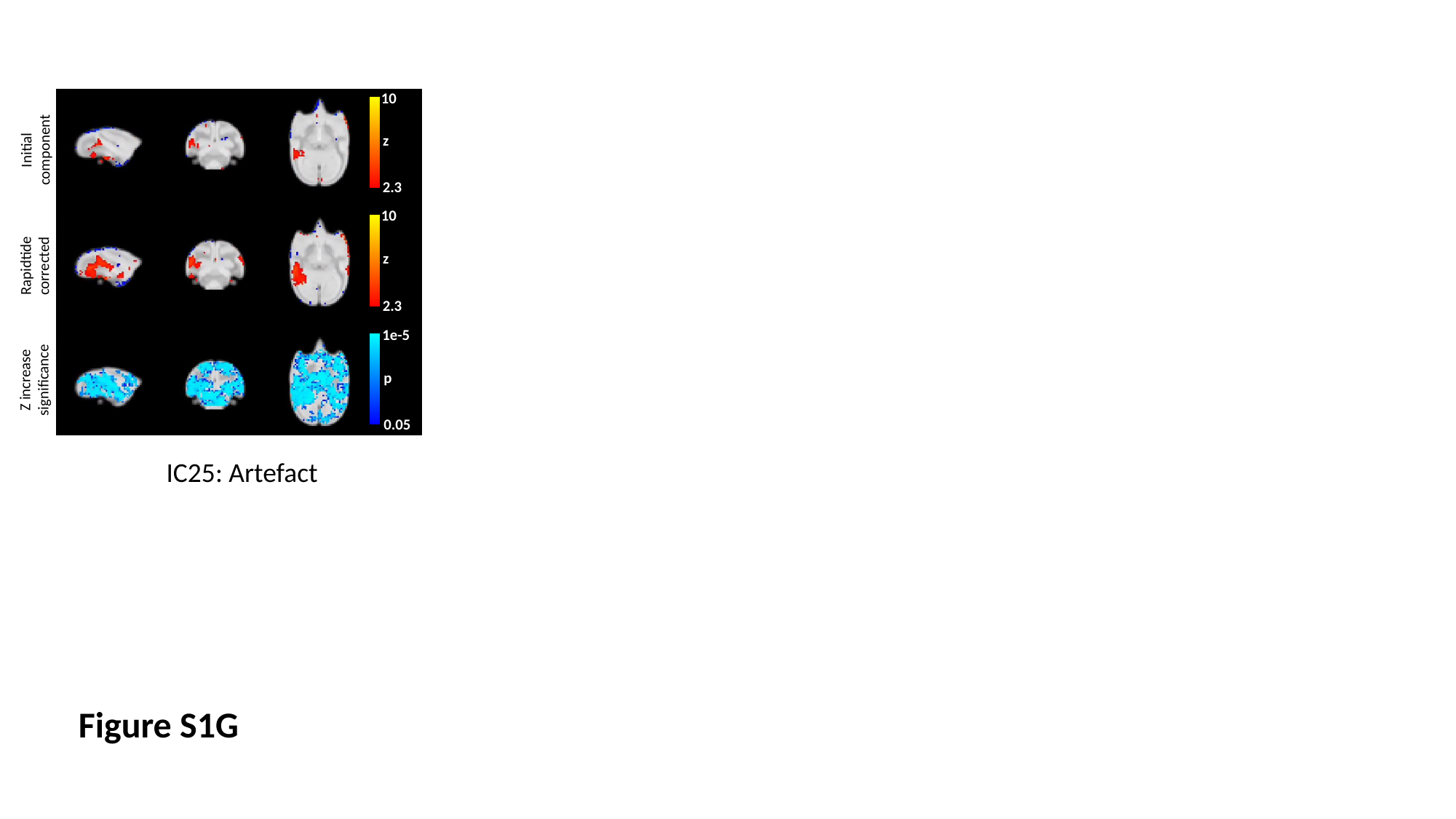

10
z
2.3
10
z
2.3
1e-5
p
0.05
Initial
component
Rapidtide
corrected
Z increase
significance
IC25: Artefact
Figure S1G

## Slide 9
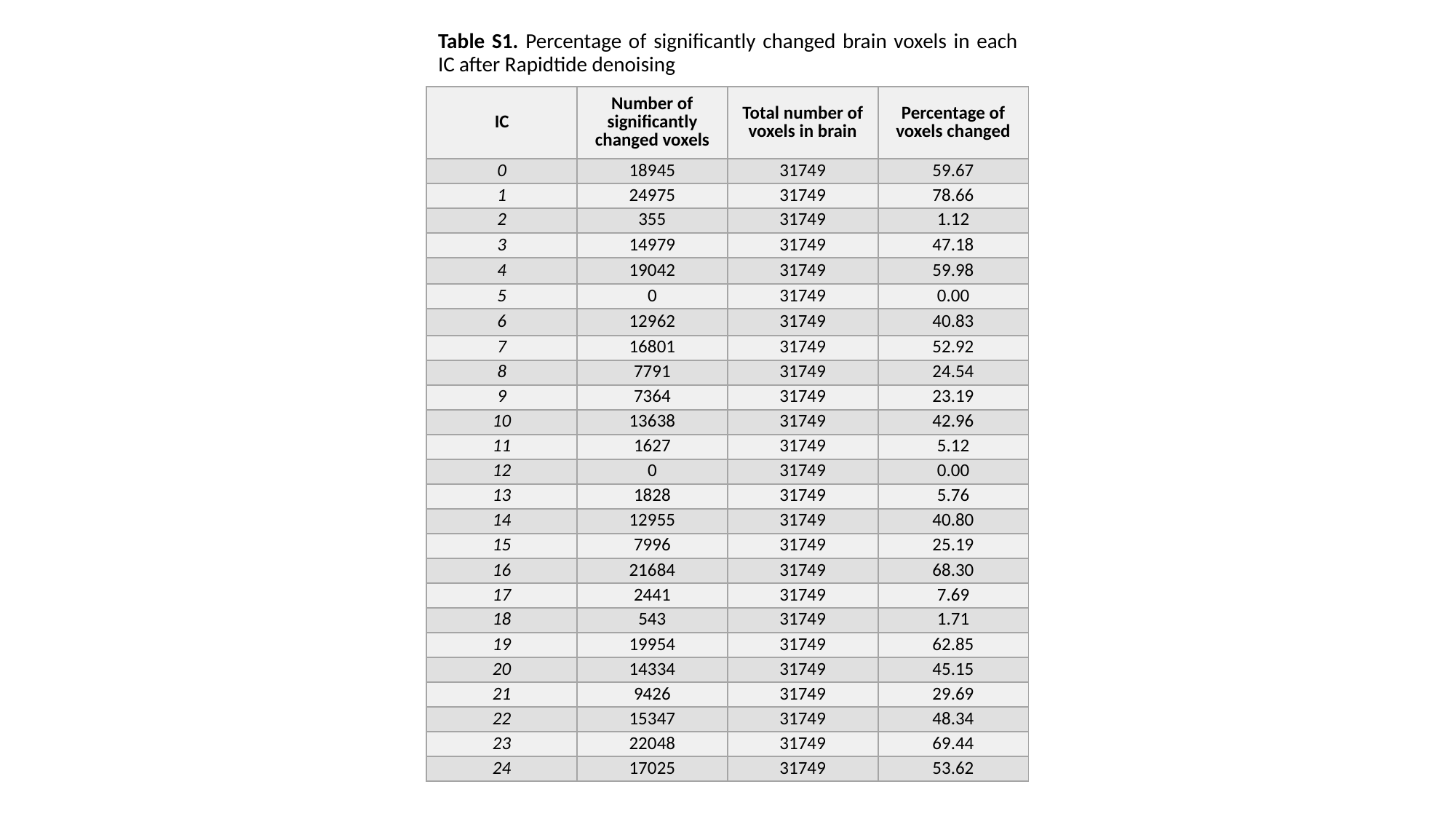

# Table S1. Percentage of significantly changed brain voxels in each IC after Rapidtide denoising
| IC | Number of significantly changed voxels | Total number of voxels in brain | Percentage of voxels changed |
| --- | --- | --- | --- |
| 0 | 18945 | 31749 | 59.67 |
| 1 | 24975 | 31749 | 78.66 |
| 2 | 355 | 31749 | 1.12 |
| 3 | 14979 | 31749 | 47.18 |
| 4 | 19042 | 31749 | 59.98 |
| 5 | 0 | 31749 | 0.00 |
| 6 | 12962 | 31749 | 40.83 |
| 7 | 16801 | 31749 | 52.92 |
| 8 | 7791 | 31749 | 24.54 |
| 9 | 7364 | 31749 | 23.19 |
| 10 | 13638 | 31749 | 42.96 |
| 11 | 1627 | 31749 | 5.12 |
| 12 | 0 | 31749 | 0.00 |
| 13 | 1828 | 31749 | 5.76 |
| 14 | 12955 | 31749 | 40.80 |
| 15 | 7996 | 31749 | 25.19 |
| 16 | 21684 | 31749 | 68.30 |
| 17 | 2441 | 31749 | 7.69 |
| 18 | 543 | 31749 | 1.71 |
| 19 | 19954 | 31749 | 62.85 |
| 20 | 14334 | 31749 | 45.15 |
| 21 | 9426 | 31749 | 29.69 |
| 22 | 15347 | 31749 | 48.34 |
| 23 | 22048 | 31749 | 69.44 |
| 24 | 17025 | 31749 | 53.62 |

## Slide 10
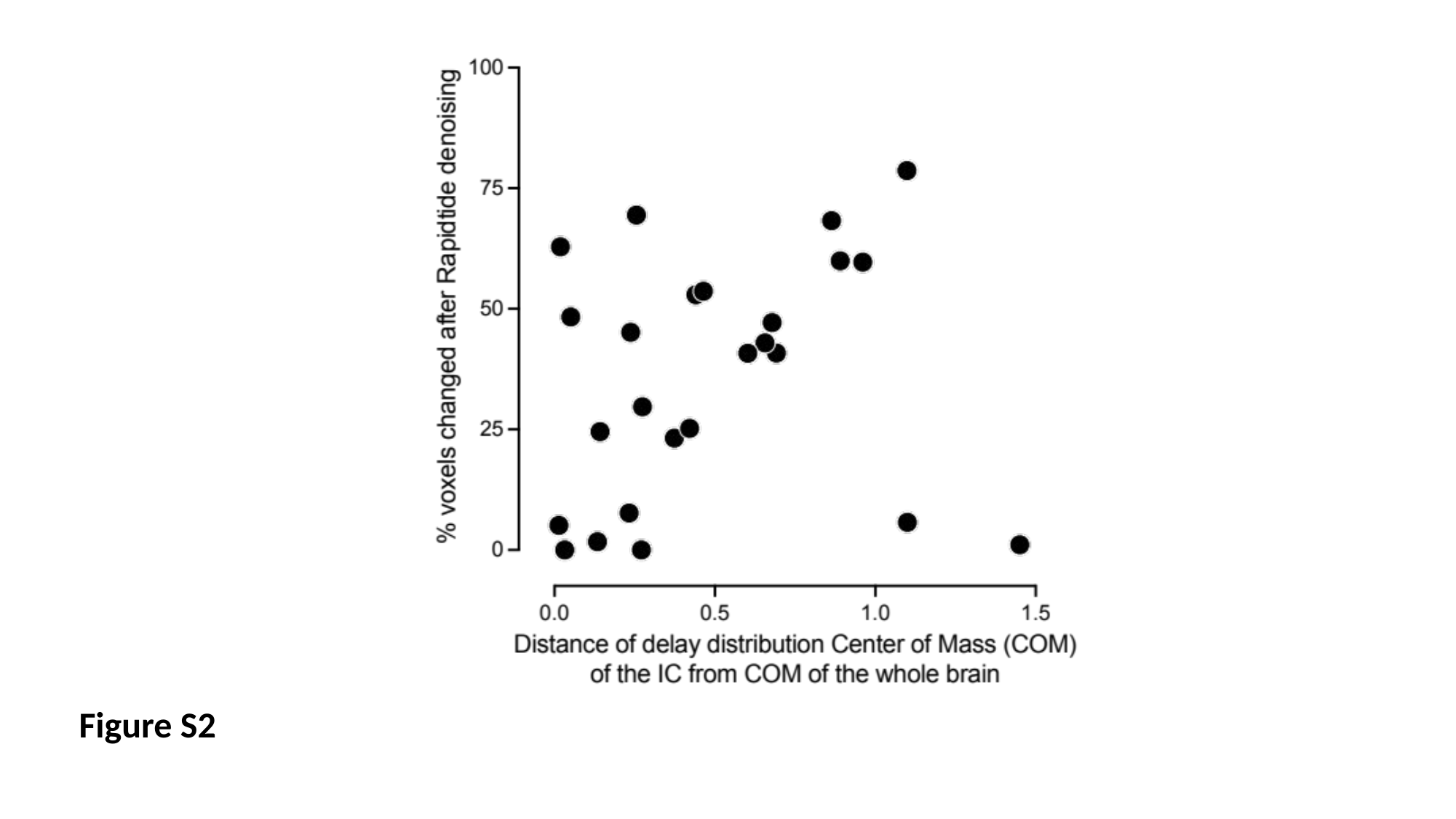

Figure S2
